# *CALN1* hypomethylation as a biomarker for high-risk bladder cancer

**DOI:** 10.1186/s12894-022-01136-y

**Published:** 2022-11-09

**Authors:** Kimiaki Takagi, Azumi Naruse, Kazutoshi Akita, Yuka Muramatsu-Maekawa, Kota Kawase, Takuya Koie, Masanobu Horie, Arizumi Kikuchi

**Affiliations:** 1 Department of Urology, Daiyukai Daiichi Hospital, 1-6-12 Hagoromo, Ichinomiya, Aichi 491-0025 Japan; 2 Department of Research and Development, Daiyukai Research Institute for Medical Science, 25 Azaicho, Ichinomiya, Aichi 491-0113 Japan; 3 Department of Urology, Gifu University Graduate School of Medicine, 1-1 Yanagido, Gifu, 501-1193 Japan

**Keywords:** Bladder cancer, *CALN1*, Methylation analysis, Methylation-sensitive restriction enzyme (MSRE), Molecular diagnosis technique, Transurethral resection of bladder tumor (TURBT)

## Abstract

**Background:**

DNA methylation in cancer is considered a diagnostic and predictive biomarker. We investigated the usefulness of the methylation status of *CALN1* as a biomarker for bladder cancer using methylation-sensitive restriction enzyme (MSRE)-quantitative polymerase chain reaction (qPCR).

**Methods:**

Eighty-two bladder cancer fresh samples were collected via transurethral resection of bladder tumors. Genomic DNA was extracted from the samples, and MSRE-qPCR was performed to determine the *CALN1* methylation percentage. Reverse transcription-qPCR was performed to assess the correlation between *CALN1* methylation and mRNA expression. The association between *CALN1* methylation percentage and clinicopathological variables of all cases and intravesical recurrence of non-muscle-invasive bladder cancer (non-MIBC) cases were analyzed.

**Results:**

Of the 82 patients, nine had MIBC and 71 had non-MIBC who had not undergone total cystectomy. The median *CALN1* methylation percentage was 79.5% (interquartile range: 51.1–92.6%). The *CALN1* methylation percentage had a negative relationship with *CALN1* mRNA expression (Spearman’s ρ = − 0.563 and *P* = 0.012). Hypomethylation of *CALN1* was associated with advanced tumor stage (*P* = 0.0007) and histologically high grade (*P* = 0.018). Furthermore, multivariate analysis revealed that *CALN1* hypomethylation was an independent risk factor for intravesical recurrence in non-MIBC patients (hazard ratio 3.83, 95% confidence interval; 1.14–13.0, *P* = 0.031).

**Conclusion:**

Our findings suggest that *CALN1* methylation percentage could be a useful molecular biomarker for bladder cancer.

**Supplementary Information:**

The online version contains supplementary material available at 10.1186/s12894-022-01136-y.

## Background

Bladder cancer is common worldwide. According to the GLOBOCAN 2018 estimates of cancer incidence and mortality, there were 549,000 new cases of bladder cancer and 200,000 associated deaths worldwide [1]. Generally, the 5-year survival rate of patients with non-muscle-invasive bladder cancer (non-MIBC) is 96%. However, if the patients have distant metastasis, the 5-year survival rate is 6% [2]. Even though non-MIBC has a relatively good prognosis, 31–78% patients with non-MIBC show recurrence and 1–45% patients show progression to MIBC within 5 years of diagnosis [3].

Cystoscopy is the most effective technique for diagnosing bladder cancer recurrence but is highly invasive. Urine cytopathology is currently widely used for diagnosis, but its sensitivity for detecting bladder cancer is low and reportedly depends on the skill of the cytopathologist [4]. Although other methods, using several biomarkers and nucleic acid probes such as bladder tumor antigen [5], nuclear matrix protein 22 [6], and UroVysion™ fluorescence in situ hybridization [7], have been developed, the robustness of these methods for the early detection of bladder cancer and risk stratification in clinical practice has not been established. Thus, there is an urgent need to establish new biomarkers.

DNA methylation is one of the epigenetic mechanisms that regulate gene expression without changing the base sequence. In recent years, DNA methylation status in bladder cancer has been widely studied [8]. Inactivation of gene expression due to promoter methylation could be a useful biomarker for bladder cancer [9–11].

We previously conducted a preliminary experiment focused on calnuelon 1 (*CALN1)*, using the Ion Ampliseq™ Methylation Panel for Cancer Research, and found that *CALN1* is associated with the clinicopathological features of bladder cancer (unpublished data). *CALN1* encodes a protein that is highly similar to the calcium-binding proteins of the calmodulin family [12]. Calcium signaling is an important regulator in various cellular processes and has been implicated in important activities related to cancer progression, such as proliferation and infiltration [13, 14]. We hypothesized that the regulation of calcium signal transduction through methylation of *CALN1* is involved in the development and progression of bladder cancer. In this study, we investigated the usefulness of determining *CALN1* methylation status as a biomarker for bladder cancer.

## Methods

### Study population

Eighty-two patients who underwent transurethral resection of bladder tumor (TURBT) between April 2019 and June 2021 at Diyukai Daiichi Hospital were enrolled in this study. Data on age; sex; presence or absence of hematuria at diagnosis; smoking status; Brinkman index; and tumor stage, grade, number, size, and type (primary/recurrent) were collected. The study was performed following approval from the Ethics Committee of the Shakai Iryo Hojin Daiyukai (approval no.2,019,002) and was conducted in accordance with the Declaration of Helsinki.

### Genomic (g)DNA isolation

Tissues collected from the patients were washed with saline and stored immediately at − 80 °C. Genomic DNA was extracted using the High Pure PCR Template Preparation Kit (Roche Molecular Systems, Pleasanton, CA, USA) according to the instruction manual, and the eluate (100 µL of elution buffer) was used for further analysis.

### Restriction enzyme treatment

The isolated DNA (100 ng gDNA) was treated with Hap II (Takara Bio, Shiga, Japan), a methylation-sensitive restriction enzyme, and/or Msp I (Takara Bio), a methylation-independent restriction enzyme, according to the manufacturer’s instruction. Hap II and Msp I are isoschizomers of each other. Hap II does not cleave the methylated recognition sequence, whereas Msp I cleaves regardless of the methylation status.

### Quantitative polymerase chain reaction (qPCR)

**Fig. 1:**
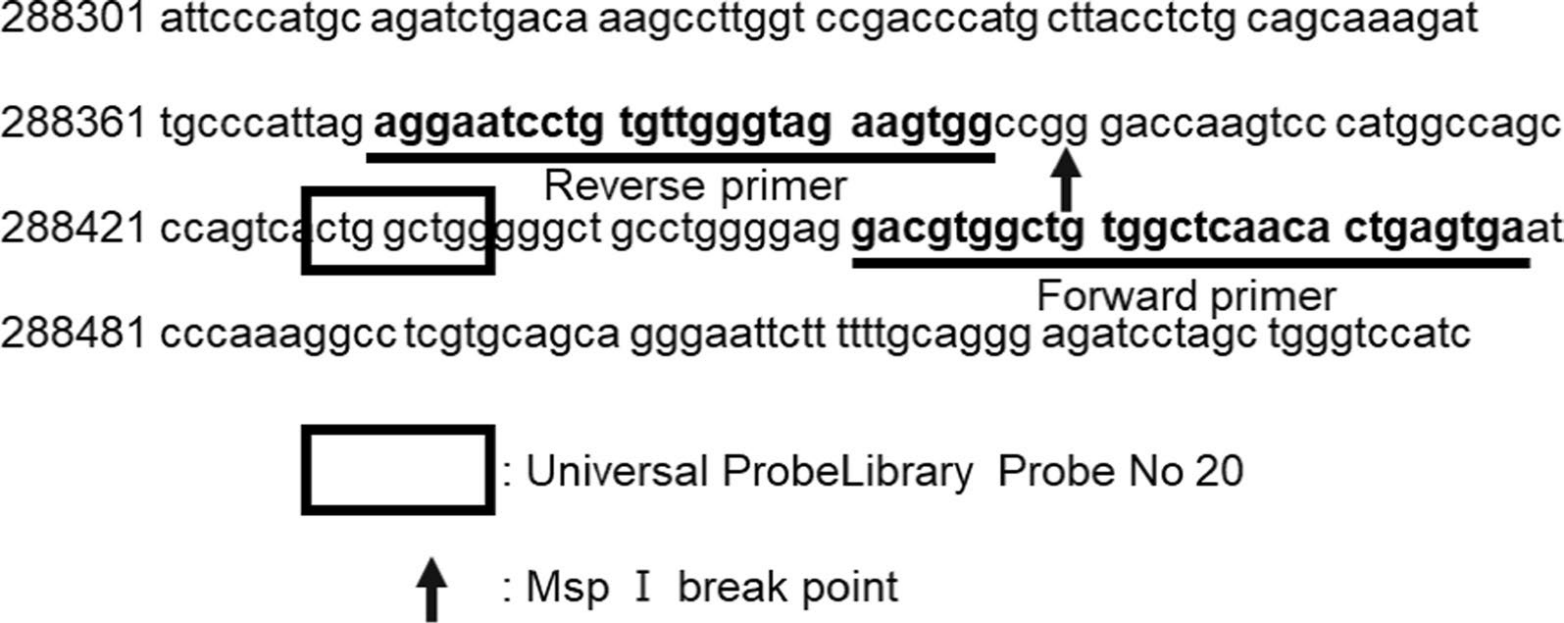
The primers were designed using the intron 2 sequence of *CALN1* with the GenBank accession no. NC_000007.14

Following enzymatic treatment, a quantitative DNA methylation analysis was performed using qPCR. Primers were designed using the intron 2 sequence of *CALN1* with the GenBank accession number NC_000007.14 (Fig. 1). The reaction was carried out in the format of a hydrolyzed probe using the following primers and probe: forward: 5′-TCACTCAGTGTTGAGCCACAG-3′, reverse: 5′-TCCTGTGTTGGGTAGAAGTGG-3′; Universal Probe Library Probes Number 20 (Roche Molecular Systems). Using a 4 µL restriction enzyme-treated gDNA solution, each primer and probe were added to 10 µL of Essential Probe Master Mix (Roche Molecular Systems) at 0.4 µM, and analysis was performed in a total volume of 20 µL. The cycling conditions included initial denaturation at 95 °C for 10 min, followed by cycles of 95 °C for 10 s, 4.4 °C/s, 60 °C for 30 s, 2.2 °C/s annealing. PCR was performed using the LightCycler 96 and data were analyzed using the LightCycler 96 software 1.1 (Roche Molecular Systems).

**Fig. 2:**
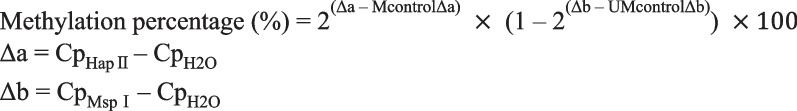
As Cp_H2O_ did not contain enzymes, amplified Cp value could be obtained regardless of methylation. On the contrary, because Cp_HapII_ was treated with HapII, which is a methylation-sensitive restriction enzyme, the amplified Cp value of only methylated sample were obtained. In addition, as Cp_MspI_ was treated with MspI, which is a methylation-independent ristriction enzyme, cleaved Cp value can be obtained regardless of methylation. The methylation percentage was calculated using Δa obtained by subtracting Cp_HapII_ from Cp_H2O_ as an index of 2, and corrected using Δb obtained by subtracting Cp_MspI_ from control gDNA or Cp_H2O_. Cp_H2O_, Cp value obtained using real time PCR analysis of sample without added enzyme; Cp_HapII_, Cp value obtained using real time PCR analysis of sample after HapII treatment; Cp_MspI_, Cp value obtained using real time PCR analysis of sample after MspI treatment

The methylation percentage was calculated using the formula shown in Fig. 2. gDNA extracted from the T24 cell line was used as the unmethylated control (UMcontrol), and EpiScope Methylated HeLa cell gDNA (Takara Bio) was used as the methylated control (Mcontrol). The nucleic acid extraction solution was adjusted to concentrations of 0, 6.25, 12.5, 25, 50, and 100% and the reaction of the measurement system was confirmed. The methylation percentage was determined from the Cp value of each sample.

### Assessment of mRNA expression via reverse transcription (RT)-qPCR

To investigate the correlation between *CALN1* methylation and mRNA expression, we performed an RT-qPCR-based assessment for the objective quantification of *CALN1* mRNA levels. Of the 82 patients, 19 who were quantitatively and qualitatively suitable for assays were used in this analysis. RNA was extracted from fresh frozen TURBT tissue using the High Pure RNA Isolation Kit (Roche Molecular Systems) according to the manufacturer’s instructions. cDNA synthesis was performed under the following reaction conditions: 25 °C for 10 min, 55 °C for 60 min, and 85 °C for 5 min. The reaction product was diluted 5-fold with TE buffer and used for subsequent reactions. Primer sequences for *CALN1* and the internal reference gene, glyceraldehyde 3-phosphate dehydrogenase (*GAPDH)*, are shown in Table 1. RT-qPCR was carried out using the LightCycler 96 (Roche Molecular Systems), and the average value of duplicate measurements was determined using the LightCycler 96 software 1.1 (Roche Molecular Systems). The comparative C(T) method in relation to *GAPDH* was used for *CALN1* expression analysis, and the correlation between *CALN1* expression and the methylation percentage was analyzed.

**Table 1:** Primer sequencing

Genes	Forward primer	Reverse primer
*CALN1*	5′-GAAGGAGTGCATTCCCAGAA-3′	5′-GCTGCAATCAGCATGACACT-3′
*GAPDH*	5′-AGCCACATCGCTCAGACA-3′	5′-GCCCAATACGACCAAATCC-3′

### Follow-up study

In our institute, cystoscopy is performed every three months after TURBT for the first two years, then every six months until five years. Intravesical recurrence of bladder cancer was defined as a tumor identified by cystoscopy and confirmed by pathological diagnosis. Intravesical BCG therapy after TURBT was performed at the discretion of the attending physician. Follow-up was conducted in November 2021. The time point of entry was defined as the date when TURBT data were obtained. The primary endpoint was the intravesical recurrence of bladder cancer.

### Statistical analyses

Because the variables were non-normally distributed, they are expressed as median and interquartile range. Differences between groups were assessed using Mann–Whitney U test. Fisher’s exact test was used to analyze categorical variables. We used Cox proportional hazards regression to examine the predictive value of *CALN1* methylation percentage for intravesical recurrence in patients with non-MIBC. The covariates included *CALN1* methylation percentage, age, sex, BCG therapy, tumor stage, tumor grade, tumor number, tumor size, and sample type (primary/recurrent). Baseline variables (P < 0.05) in the univariate analysis were included in the multivariate models. A receiver operating characteristic (ROC) curve was generated, and the area under the curve was calculated to determine the appropriate cut-off level of *CALN1* methylation percentage to maximize the predictive power for intravesical recurrence-free survival of patients with non-MIBC. The methylation percentage was grouped into low and high based on the cut-off value confirmed using the ROC curve analysis. Kaplan–Meier curves of estimated intravesical recurrence-free survival were generated, and comparisons between the groups were performed using a 2-sided log-rank test.

To assess whether the accuracy of predicting intravesical recurrence would improve after the addition of *CALN1* methylation percentage to established risk factors, including tumor stage, grade, number, size, and sample type, we calculated the C-index, net reclassification improvement, and integrated discrimination improvement. The *CALN1* methylation percentage and value of mRNA expression were not normally distributed (assessed using the Shapiro–Wilk test); therefore, non-parametric correlation coefficients (Spearman’s [ρ]) were used to determine the association between *CALN1* methylation percentage and mRNA expression. Statistical significance was set at P < 0.05 and all statistical tests were two-sided. Statistical analyses were performed using the R software version 4.0.3.

## Results

In total, 82 patients (MIBC, n = 9; non–MIBC, n = 73) were enrolled in this study. During the follow-up period (median, 11.5 months), 13 of the 82 patietns died. Twenty-five of the remaining 71 patients whose bladders were preserved showed intravesical recurrence within 1 year. Of these 25 patients, 6 patients died. Three of the six patients died of bladder cancer. The median *CALN1* methylation percentage was 79.5% (interquartile range: 51.1–92.6). In the univariate Cox proportional hazards analysis, the *CALN1* methylation percentage was a significant predictor of intravesical recurrence (hazard ratio (HR) 0.98, 95% confidence interval (CI) 0.97–1.00, *P* = 0.0010). After adjusting for other confounders, the *CALN1* methylation percentage was an independent predictor of intravesical recurrence (HR 0.98, 95% CI 0.97–1.00, *P* = 0.018). The ROC analysis was performed to maximize the predictive power of *CALN1* methylation percentage for intravesical recurrence, and an 87% cut-off value was obtained (area under the curve = 0.711). Based on the cut-off value, 82 patients fit into two groups. Fifty-one (62%) patients fit in the low group with a methylation percentage of less than 87%, and 31 (38%) fit in the high group with a methylation percentage greater than 87%. Patient characteristics according to *CALN1* methylation percentage are shown in Table 2 (Additional file 1). Of the 82 patients, 73 had non-MIBC and 9 had MIBC. Total cystectomy was performed in two patients with non-MIBC that was difficult to cure by TURBT during the follow-up period. Patients in the low group were significantly older than those in the high group and had a higher proportion of females and non-smokers. In addition, the low group tended to have significantly advanced tumor stages and more histologically high-grade tumors than the high group.

**Table 2:** Clinicopathological features according to *CALN1* methylation percentage

Variable		All n = 82	*CALN1* methylation		*P*
			Low n = 51	High n = 31	
*CALN1* methylation percentage (IQR^a^)		79.5 (51.1, 92.6)	63.1 (40.7, 78.1)	97.8 (90.6, 100)	< 0.0001
Follow-up period, month (range)		11.5 (0–29)	12 (0–29)	11 (0–29)	0.21
Age (range)		76 (52–93)	79 (53–93)	73 (52–85)	< 0.0001
Sex	Female, n (%)	19 (23)	17 (33)	2 (6)	0.0062
Hematuria	Yes, n (%)	39 (48)	24 (47)	15 (48)	0.91
Smoking history	Yes, n (%)	45 (55)	23 (45)	22 (71)	0.039
Brinkman index	(IQR)	200 (0–700)	0 (0–490)	640 (0–990)	0.0073
BCG^b^ therapy	Yes, n (%)	11 (13)	7 (13)	4 (13)	1
Total cystectomy		9 (11)	5 (10)	4 (13)	0.72
Tumor stage, n (%)					0.0007
Ta		54 (66)	27 (53)	27 (87)	
T1		19 (23)	18 (35)	1 (3)	
T2+		9 (11)	6 (12)	3 (10)	
Tumor grade, n (%)					0.018
Low		62 (76)	34 (67)	28 (90)	
High		20 (24)	17 (33)	3 (10)	
Tumor number, n (%)					0.65
Single		41 (50)	24 (47)	17 (55)	
Multiple		41 (50)	27 (53)	14 (45)	
Tumor size, n (%)					0.12
< 30 mm		70 (85)	41 (80)	29 (94)	
≥ 30 mm		12 (15)	10 (20)	2 (6)	
Sample type, n (%)					0.65
Primary		50 (61)	30 (59)	20 (65)	
Recurrent		32 (39)	21 (41)	11 (35)	

^a^* IQR* Interquartile range; ^b^* BCG* Bacillus Calmette-Guérin

**Fig. 3:**
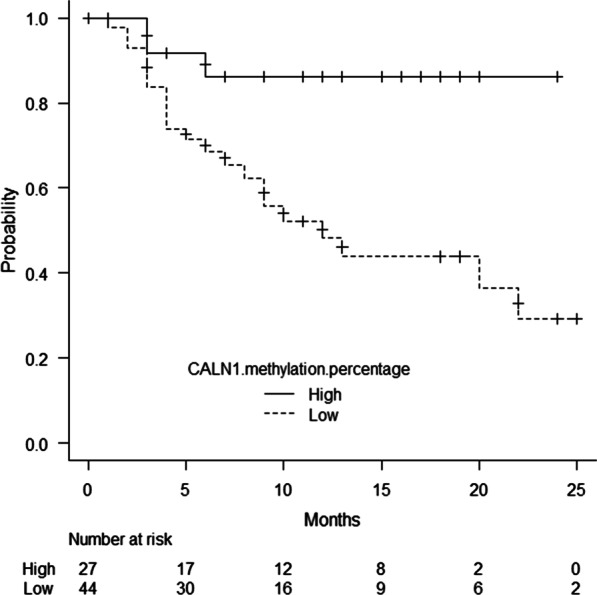
Kaplan-Meier curves of intravesical recurrence-free survival of patients with non-MIBC in high and low *CALN1* methylation percentages groups

**Fig. 4:**
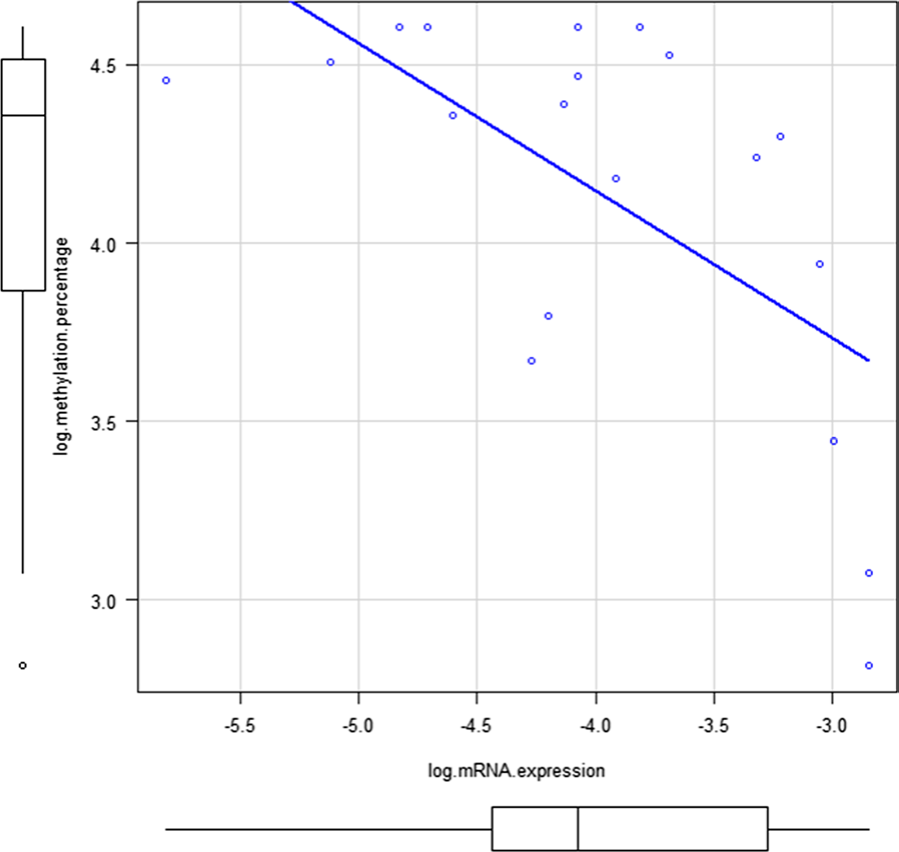
*CALN1* methylation percentage has a negative relationship with *CALN1* mRNA expression (Spearman’s ρ = − 0.563 and *P* = 0.0121)

To identify the association between the *CALN1* methylation percentage and intravesical recurrence, a Kaplan–Meier analysis was performed in 71 patients with non-MIBC whose bladders were preserved. There was a significant difference between the groups in terms of intravesical recurrence-free survival (*P* = 0.0084). At the one-year follow-up, the Kaplan–Meier survival rates of patients with intravesical recurrence were 48.2% and 86.3% in the low and high groups, respectively (Fig. 3). The results of the univariate and multivariate Cox regression analyses to explore the prognostic factors of intravesical recurrence are shown in Table 3. A low *CALN1* methylation percentage remained an independent prognostic factor after adjusting for tumor size in the multivariate analysis. The C-index increased, but did not reach statistical significance (0.744, *P* = 0.27). However, the net reclassification improvement and integrated discrimination improvement for the intravesical recurrence rate significantly improved after adding the *CALN1* methylation percentage to the baseline model with established risk factors (0.57 and 0.07, *P* = 0.021 and *P* = 0.025, respectively, Table 4). In the analysis of the correlation between *CALN1* methylation and the mRNA expression level, a significant negative correlation was observed (Fig. 4, Additional file 2).

**Table 3:** Prognostic value of CALN1 methylation percentage for intravesical recurrence of bladder cancer

Variables	Univariate		Multivariate	
	HR^a^ (95% CI^b^)	*P*	HR (95% CI)	*P*
*CALN1* methylation percentage	0.98 (0.97–1.00)	0.011	0.98 (0.97–1.00)	0.018
Age	1.03 (0.99–1.09)	0.15		
Sex (male)	0.63 (0.25–1.60)	0.33		
Smoking history (yes)	0.50 (0.23–1.11)	0.087		
BCG^c^ therapy (yes)	0.41 (0.10–1.75)	0.23		
Stage (Ta)	0.56 (0.24–1.32)	0.19		
Grade (low)	0.63 (0.25–1.61)	0.34		
Number (single)	1.02 (0.47–2.26)	0.95		
Size (≥ 30 mm)	4.25 (1.50–12.1)	0.0065	3.75 (1.33–10.6)	0.012
Recurrent tumor	0.84 (0.38–1.88)	0.68		

^a^* HR* Hazard ratio, ^b^* CI* Confidence interval, ^c^* BCG* Bacillus Calmette-Guérin

**Table 4:** Discrimination of each predictive model for intravesical recurrence using C-index, net reclassification improvement (NRI), and integrated discrimination improvement (IDI)

Predictive models	C-index	P	NRI	P	IDI	P
Established risk factors^a^	0.67 (0.53–0.82)	Reference	Reference		Reference	
+*CALN1* methylation percentage	0.74 (0.61–0.87)	0.27	0.57	0.021	0.07	0.025

^a^ Established risk factors included tumor stage, grade, number, size, and sample type

## Discussion

We analyzed the relationship between the *CALN1* methylation percentage and clinicopathological data of patients with bladder cancer. We found that *CALN1* hypomethylation was significantly associated with advanced tumor stage, more histologically higher-grade tumors, and an increased risk of intravesical recurrence. To the best of our knowledge, this is the first study to show that *CALN1* methylation percentage is associated with the clinicopathological features and prognosis of bladder cancer.

The association between DNA methylation and various biological phenomena such as carcinogenesis have been identified [15, 16]. Methylation analysis could provide information that cannot be obtained using conventional tests, such as prediction of drug sensitivity or prognosis [17, 18].

Cao et al. used microarray analysis to show that calcium signal transduction was associated with the development of bladder cancer via the mitogen-activated protein kinase pathway [19]. In addition, intron 2 of *CALN1* is a DNase I hypersensitive site that is strongly associated with transcriptional activity [20]. Therefore, we suspected that *CALN1* methylation was involved in the action of a DNase I hypersensitive site and, as a result, may affect the expression of *CALN1*. Regarding the relationship between bladder cancer and methylation, various analytical reports have centered on CpG sites [21, 22], and testing systems such as Bladder EpiCheck [23] have been established. Although various trials have been conducted regarding the diagnosis and treatment of bladder cancer, methylation analysis of *CALN1* and its association with bladder cancer has not been probed before.

Bisulfite sequencing is widely used for methylation analyses. In this study, we performed methylation analysis using methylation-sensitive restriction enzyme (MSRE)-qPCR. This technique enables the analysis of a small amount of sample obtained by TURBT without bisulfite treatment. Bisulfite treatment involves the process of incubating the DNA solution at 50–70 ℃. There is a problem that the yield of DNA is extremely low because the DNA is cleaved during the heating process. Recently, high-yield methods have been developed, but DNA fragmentation has not been avoided completely [24]. In addition, because bisulfite sequencing requires a large number of cells, it is not feasible for clinical specimens with a low amount of DNA such as cell-free DNA or circulating tumor cells. In contrast, one of the advantages of MSRE-qPCR is the side-by-side comparison between control and experimental samples, even for very low amounts of DNA. In addition, MSRE-qPCR can be completed in less time than other methods with the same level of accuracy [25]. Comprehensive analysis using next-generation sequencing is also useful but less practical owing to high costs. MSRE-qPCR is useful for targeted analysis owing to its simple workflow. Further investigation exploring this diagnostic method with high sensitivity and specificity in combination with other diagnostic markers is necessary and will contribute to the development of new diagnostic systems for bladder cancer.

The current study has some limitations. First, there were no criteria for intravesical BCG immunotherapy, though there was no difference in BCG therapy between the low- and high-methylation groups. Second, required sample size was not calculated before the study. However, based on the results obtained, the required sample size for comparison of the survival curves between the groups was calculated to be 20 patients per group. The sample size of this study was sufficient to meet this requirement. Nevertheless, the sample size was small and the follow-up period was short. Therefore, the findings of this study need to be validated in a larger study.

## Conclusion

We performed methylation analysis of intron 2 of *CALN1* using gDNA extracted from samples collected by TURBT. We found that low *CALN1* methylation percentage is consistent with the occurrence of advanced tumor stages, high-grade tumors, and higher intravesical recurrence rates. Therefore, we suggest that *CALN1* methylation percentage may be an indicator of high-risk bladder cancer and could be considered a useful biomarker for accurately predicting intravesical recurrence of non-MIBC.

## Supplementary Information

**Additional file 1.** Demographicaland clinical information of the subjectsparticipating in this study.

**Additional file 2.** Raw dataof relationship between CALN1 mRNA expression and CALN1 methylation percentage.

## Data Availability

All data generated or analyzed during this study are included in this published article and its supplementary information files.

## Abbreviations

MSRE: Methylation-sensitive restriction enzyme
qPCR: Quantitative polymerase chain reaction
MIBC: Muscle-invasive bladder cancer
TURBT: Transurethral resection of bladder tumor
gDNA: Genomic DNA
RT-qPCR: Reverse transcription-qPCR
GAPDH: Glyceraldehyde 3-phosphate dehydrogenase
ROC: Receiver operating characteristic
HR: Hazard ratio
CI: Confidence interval
